# Deep Learning for Improved Risk Prediction in Surgical Outcomes

**DOI:** 10.1038/s41598-020-62971-3

**Published:** 2020-06-09

**Authors:** Ali Jalali, Hannah Lonsdale, Nhue Do, Jacquelin Peck, Monesha Gupta, Shelby Kutty, Sharon R. Ghazarian, Jeffrey P. Jacobs, Mohamed Rehman, Luis M. Ahumada

**Affiliations:** 1 Predictive Analytics, Johns Hopkins All Children’s Hospital, St. Petersburg, FL 33701 USA; 2 Department of Anesthesia and Pain Medicine at Johns Hopkins All Children’s Hospital, St. Petersburg, FL 33701 USA; 3 Pediatric Cardiac Surgery, Department of Surgery at Vanderbilt University, Nashville, TN 37240 USA; 4 Department of Anesthesiology at Mount Sinai Hospital, Miami Beach, FL 33140 USA; 5 Division of Cardiology at Johns Hopkins All Children’s Hospital, St. Petersburg, FL 33701 USA; 6 Department of Pediatrics, at Johns Hopkins School of Medicine, Baltimore, MD 21287 USA; 7 Health Informatics Core, Johns Hopkins All Children’s Hospital, St. Petersburg, FL 33701 USA; 8 Unaffiliated, St. Petersburg, FL USA

**Keywords:** Translational research, Risk factors, Information technology

## Abstract

The Norwood surgical procedure restores functional systemic circulation in neonatal patients with single ventricle congenital heart defects, but this complex procedure carries a high mortality rate. In this study we address the need to provide an accurate patient specific risk prediction for one-year postoperative mortality or cardiac transplantation and prolonged length of hospital stay with the purpose of assisting clinicians and patients’ families in the preoperative decision making process. Currently available risk prediction models either do not provide patient specific risk factors or only predict in-hospital mortality rates. We apply machine learning models to predict and calculate individual patient risk for mortality and prolonged length of stay using the Pediatric Heart Network Single Ventricle Reconstruction trial dataset. We applied a Markov Chain Monte-Carlo simulation method to impute missing data and then fed the selected variables to multiple machine learning models. The individual risk of mortality or cardiac transplantation calculation produced by our deep neural network model demonstrated 89 ± 4% accuracy and 0.95 ± 0.02 area under the receiver operating characteristic curve (AUROC). The C-statistics results for prediction of prolonged length of stay were 85 ± 3% accuracy and AUROC 0.94 ± 0.04. These predictive models and calculator may help to inform clinical and organizational decision making.

## Introduction

Hypoplastic left heart syndrome (HLHS) is one of several severe congenital cardiac defects involving a single ventricle physiology. HLHS is caused by under-development or non-development of the aortic or mitral valves, so blood lacks a physical outlet to the systemic circulation, leaving only a rudimentary left ventricle. The right side of the heart develops intact and therefore only the right ventricle is an effective pump for blood^1^.

Definitive management, without which the child will die, consists of cardiac surgery in the form of a Norwood procedure within the first few days of life. This is a complex surgical procedure that transitions blood flow from parallel pulmonary and systemic circulations to restore sequential blood flow by using either a modified Blalock Taussig shunt or a right ventricle to pulmonary artery (RV-to-PA) (Sano) shunt^2,3^. As the child grows, further stages of surgery are required to optimize the circulation.

The incidence of HLHS is approximately 1 in 5000 live births^4^, or aproximately 750 births per year in the USA^5^. Despite advances in care, mortality rates among infants born with HLHS remain high, reported at 15–20%^6^. To evaluate patient outcomes following repair of single ventricle congenital malformations such as HLHS, the Pediatric Heart Network (PHN) conducted the Single Ventricle Reconstruction (SVR) Trial. The PHN SVR trial incorporated 555 infants who were treated at one or more of 15 participating medical centers from 2005 to 2009. Data were recorded at various stages throughout the course of treatment and included evaluations at baseline (pre-operative), at the time of the Norwood procedure, before second stage surgical procedures (inter-procedure), and post-operatively to age 14 months^7,8^.

Existing single ventricle physiology risk models focus on patient mortality and do not yet assess the risk for prolonged length of hospital stay. Since neonatal cardiac surgery is emotionally taxing, a risk assessment for prolonged length of stay may benefit these families by guiding expectations. Additionally, with the increasing national emphasis on efficient medical practice and standardization of care, surgeons, operating room staff, and hospital administrators are in need of a way to predict comorbidities, adverse events and length of stay for hospital patients. Such information is helpful in optimization of resource utilization and is used for prioritizing quality improvement.

Deidentified results from the PHN SVR trial are publicly available for research. Independent groups including Tabbutt *et al*., Chowdhury *et al*. and Gupta *et al*. have developed risk prediction models derived from PHN SVR data. Tabbutt *et al*.^9^, analyzed time to death following hospital discharge after Norwood procedure using Kaplan-Meier estimation and Cox proportional hazards regression. They concluded that factors such as the use of Extra Corporeal Membrane Oxygenation and center/surgeon volume are important in predicting the postoperative risk of mortality limited to Norwood hospitalization, but they did not provide patient specific risk of mortality. Chowdhury *et al*.^10^ developed a composite score to assess the risk of in-hospital mortality for these patients. They used logistic regression modeling technique on six variables to develop their scoring system. Their results showed ${R}^{2}=0.82$ and area under the receiver operating characteristic curve (AUROC) of 0.79 for the test data. Gupta *et al*.^11^ used Bayesian conditional probability regression and Markov Chain-Monte Carlo (MCMC) simulations to create a predictive model and calculator. Although they reached an overall accuracy of 75%, the predictive model they produced required intraoperative data values and so could not be used for preoperative decision making. Our study aims to address the need to provide an accurate patient specific risk score for long-term (one-year) postoperative mortality or cardiac transplantation and prolonged length of hospital stay, based on the data available at the time the prediction would be most clinically useful. The primary purpose for providing this information is to assist clinicians and patients’ families in the decision to proceed down the high-risk surgical treatment route beginning with Norwood procedure and carrying the only chance of long term survival. The alternative to surgical correction is comfort care leading to early death. In the cases with a very high risk of mortality, this may be agreed upon by clinicians and the patient’s family as the most appropriate course of action.

Many existing risk models within adult populations assess prolonged length of stay (LOS) in the intensive care unit (ICU) following cardiac surgery. A prolonged LOS occurs because the patient has a complicated postoperative recovery. This may be due to one of many factors, including a more fragile preoperative state or the occurrence of postoperative complications such as infections or bleeding. As well as a greater emotional strain on the patient’s family, it is associated with increased healthcare costs and resource utilization compared with an uncomplicated recovery.

Tu *et al*. applied a neural network model with 15 pre-operative factors to predict prolonged length of stay in the ICU following adult cardiac surgery^12,13^. Similarly, Widyastuti *et al*.^14^ use a Cox proportional hazards regression model to predict prolonged LOS following cardiac surgery in adults. After reviewing 29 different studies for ICU LOS prediction, Almashrafi *et al*.^15^ concluded that LOS increases with increasing age and growing number of comorbidities. Ettema *et al*.^16^ compared 14 models of prolonged ICU LOS prediction and concluded that the best model has an area under the characteristic operating curve (AUROC) of 0.75; although this result is acceptable for clinical use it still has room for improvement. These existing models are also not yet validated by controlled, multi-institutional studies^17^.The studies in adults each defined prolonged LOS differently and all within the range 2–7 days. This range is inappropriate for the Norwood procedure as all patients will be expected to stay in hospital for longer than 7 days. Few LOS models have been developed for children or infants undergoing cardiac surgery, although the pediatric heart network investigators^18,19^ have reported on risk factors for prolonged length of stay following the Glenn and Fontan procedures. Currently, there is no precise definition of *prolonged length of stay* in the literature, but previous studies using the PHN SVR dataset have used a threshold at the 75th percentile^20,21^. Schwartz *et al*.^18^ defined prolonged LOS after Norwood as the median length of stay for transplant-free survivors who later underwent Glenn procedure. By definition this median will produce a group containing 50% of the population and is therefore not sensitive enough for clinical use such as parental counseling and resource planning.

In this study, we apply machine learning based classification to investigate patient specific outcomes after Norwood procedure. We use the 549 cases who were randomized in the PHN SVR trial to develop a new predictive model and precision medicine calculator to show individualized risk of prolonged LOS and risk of mortality or cardiac transplantation at one year after the operation. Through this analysis we will better inform preoperative risk assessment and shared decision making by use of data-driven precision medicine.

## Methods

**Figure 1:**
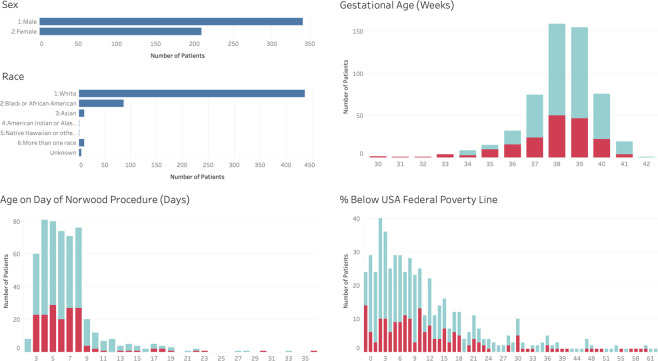
Demographics of patients in the PHN SVR dataset. The patient’s sex, race and age on the day of their Norwood surgical procedure is shown. Gestational age is the gestational age at birth reported in weeks, indicating presence and degree of prematurity with full term ≥ 37 weeks. % below federal poverty level is an indication of socioeconomic status. The turquoise color represents patients who survived to one year, red represents those who died.

The PHN SVR Trial investigators collected data at 15 centers from 2005 to 2009. The trial screened a total of 920 newborns with single ventricle physiology. Of these patients, 664 met inclusion criteria and 555 were randomized to undergo either a MBTS or a RV-to-PA shunt during the Norwood procedure. Six patients dropped out of the protocol during the trial and 189 patients did not survive the first year^22,23^. The data utilized for prediction of risk of mortality comprise the 549 patients who completed the study. We defined LOS as per Schwartz *et al*.^18^ and therefore our dataset for prediction of risk of prolonged length of stay comprise the 477 patients who survived to transplant-free hospital discharge. Figure 1 summarizes the demographics of the patients in the dataset.

All analyses were performed in accordance with relevant guidelines and regulations. The study protocol was prospectively approved by the Johns Hopkins All Children’s Hospital Institutional Review Board for the inclusion of children as ‘research not involving greater than minimal risk’. The permission of parents/guardians and assent of children was waived (IRB00143503).

Using the PHN SVR trial data, we developed and tested multiple machine learning algorithms to predict the individualized risk of one-year mortality or cardiac transplantation and prolonged hospital length of stay for patients undergoing the Norwood procedure. The algorithm design consisted of three main stages: data pre-processing, model building and model evaluation and validation.

### Data pre-processing

#### Data scaling

Data scaling is an essential part of machine learning modeling as those variables with large values tend to have a greater influence on prediction error and hence will have higher weight. In order to overcome this problem we used a softmax scaling algorithm to normalize numerical data. Softmax scaling is a nonlinear transformation that maps the data into the range of $[0,1]$. The softmax scaling consists of two steps:

1 $$d=\frac{{x}_{ik}-{\bar{x}}_{k}}{{\sigma }_{k}}$$

2 $${\hat{x}}_{ik}=\frac{1}{1+\exp (\,-\,d)}$$

where ${x}_{ik}$ is *i*th value of *k*th variable, ${\bar{x}}_{k}$ is mean of *k*th variable, ${\sigma }_{k}$ is variance of *k*th variable, and d is the dummy transformation variable. An advantage of softmax over other scaling methods is that it will transform the data distribution into a Gaussian distribution, which is helpful in many machine learning algorithms and specifically with Markov Chain-Monte Carlo (MCMC) multiple imputation (MI) method since it guarantees multivariate normality. We also used min-max scaling technique to scale categorical and ordinal data. Min-max scaling rescales the data to the range of $[\,-\,1,1]$ to ensure that weights of the model are not biased to any model inputs.

#### Missing data

Machine learning algorithms will not perform as intended if the dataset contains missing values. Therefore any missing values or gaps must be imputed using advanced missing data techniques such as multiple imputation. In the method of mutiple imputation we create $m$ imputations that are intended to represent a probable range of values to approximate the missing data. The resulting variability of values enables us to quantifiy the uncertainty in the imputation process and integrate it into our analysis^24^. The most frequent missing values from the PHN SVR trial dataset were highest serum lactate (18% missing), poverty score (6% missing), and APGAR scores at 1 and 5 minutes (5% missing). To handle this missing data, we performed MCMC MI under the assumption of multivariate normality^25,26^. Following the analysis and assumptions made by the investigators of the PHN-SVR trail^27^ we assumed that the data is missing at random (MAR) which means that the probability of data missing for a variable depends only on other variables within the dataset.

MCMC is an iterative method that repeats two steps: the imputation step and the posterior step. In the imputation step at time $t$, we draw values for missing observations ${X}_{miss}^{t+1}$ from a conditional distribution $p({X}_{miss|{X}_{obser},{\zeta }^{t}})$ where, ${X}_{obser}$ are observed values and $\zeta$ are Gaussian distribution parameters. In the posterior step, we draw ${\zeta }^{t+1}$ from conditional probability $p(\zeta |{X}_{obser},{X}_{miss}^{t+1})$ which creates a Markov chain $({X}_{miss}^{1},{\zeta }^{1})$,$({X}_{miss}^{2},{\zeta }^{2})$, …, $({X}_{miss}^{n},{\zeta }^{n})$^28^.

We first randomly divided the dataset into training (70%) and testing (30%) sets and then used the multiple imputation procedure command (PROC MI) in SAS software^29^ to separately create 50 datasets for each of the training and testing sets. This separation helped to ensure that the test set was kept completely independent from the training set. Studies have shown that dividing the data before MI procedures leads to reduced bias when estimating performance metrics^30,31^. This approach was possible because we had access to the complete test set before performing MI methodology. To create each new dataset, the software used the MCMC method to impute the missing values ${X}_{miss}^{2}$, and updated the distribution function ${\zeta }^{i}$. Fifty times imputation has been shown to produce the narrowest confidence intervals in datasets with higher missing value rates^32^. We then could analyze each of these imputed datasets using machine learning modeling methods and estimate performance measures. Differences in the values during multiple imputation cause these estimates to vary, hence these estimates should be pooled to generate the final estimates of the performance measures and standard error bounds for these estimates. The variance of the overall estimate is a function of variance within each imputed dataset and cross dataset’s variance^33^.

### Model building and training

#### Variable selection

In a similar method to previous studies^9,11,18,19,34^, we used established clinical importance and clinical expert opinion, to select 25 out of more than 100 preoperative variables from the PHN SVR dataset as inputs to the algorithms for calculating risk of one-year mortality or cardiac transplantation. These are presented in Table 1. We used only preoperative variables for mortality or cardiac transplantation prediction since the purpose of the model is to provide detailed information for decision making and family counseling before surgery.

**Table 1:** Selected variables from the dataset for machine learning modeling.

Category	Variables	Description	Scaling
Preoperative	Age	Days	softmax
	Sex	Male/Female	None
	Low birth weight	Birth weight < 2500g	None
	Poverty score	Percentage income below federal poverty level	softmax
	Race	Race of the patient	softmax
	Surgery Volume	Number of Norwood surgeries at the hospital/year	min-max
	Gestational age at birth	Weeks	softmax
	Pre-term	Yes/No	None
	Prenatal diagnosis of congenital heart disease	Yes/No	None
	Fetal age at prenatal diagnosis	Weeks	softmax
	Any associated anatomic diagnoses?	Yes/No	None
	Apgar 1	Apgar score at 1 minute	min-max
	Apgar 5	Apgar score at 5 minutes	min-max
	Highest serum lactate	mmol/L	softmax
	Inhaled *CO*_2_	Yes/No	None
	Inhaled *N*_2_	Yes/No	None
	Anatomic diagnosis	HLHS, Transposition of great arteries (TGA), etc.	min-max
	Pre-surgery cardiac catheterization?	Yes/No	None
	Fetal interventions	Yes/No	None
	Aortic Atresia	Yes/No	None
	Obstructed pulmonary venous return?	Yes/No	None
	Presence of HLHS?	Yes/No	None
	Number of significant pre-operative complications	0-5	min-max
	Number of pre-Norwood surgical interventions	0-4	min-max
	Type of shunt	Blalock Taussig or RV-to-PA	None
Intraoperative	Treatment	MBTS or RV-to-PA	None
	Cross-Clamp time	Minutes	softmax
	Bypass time	Minutes	softmax
	DHCA time	Deep Hypothermic Circulatory Arrest time in minutes	softmax
	RCP time	Retrograde Cerebral Perfusion time in minutes	softmax
	RCP flow	Retrograde Cerebral Perfusion cc/Kg/min	softmax
	Low Temp	Lowest temperature °C	softmax
	Lowest Hematocrit	_%_	softmax
	Ultrafiltration used during Cardiopulmonary Bypass (CPB)?	Yes/No	None
	Ultrafiltration used post CPB?	Yes/No	None
	Steroids given intraoperatively	Yes/No	None
	Trasylol (Aprotinin) given intra-operatively	Yes/No	None
	Alpha adrenergic receptor blockade?	Yes/No	None
	Was patient placed on extracorporeal membrane oxygenation?	Yes/No	None
	Exterior diameter ascending aorta	mm	softmax
	Type of arch reconstruction	Classic or direct	None
	Coarctectomy	Yes/No	None
	MBTS diameter	mm	softmax
	MBTS length	mm	softmax
	RV-to-PA diameter	mm	softmax
	RV-to-PA length	mm	softmax
	Was patient extubated in the operating room?	Yes/No	None
	Did patient require cardiopulmonary resuscitation?	Yes/No	None
	Oxygen saturation at the end of surgery	_%_	softmax

Preoperative data was used for mortality prediction model, while a combination of both preoperative and intraoperative data was only used for prolonged LOS prediction. Scaling shows the methodology used to scale the data.

**Figure 2:**
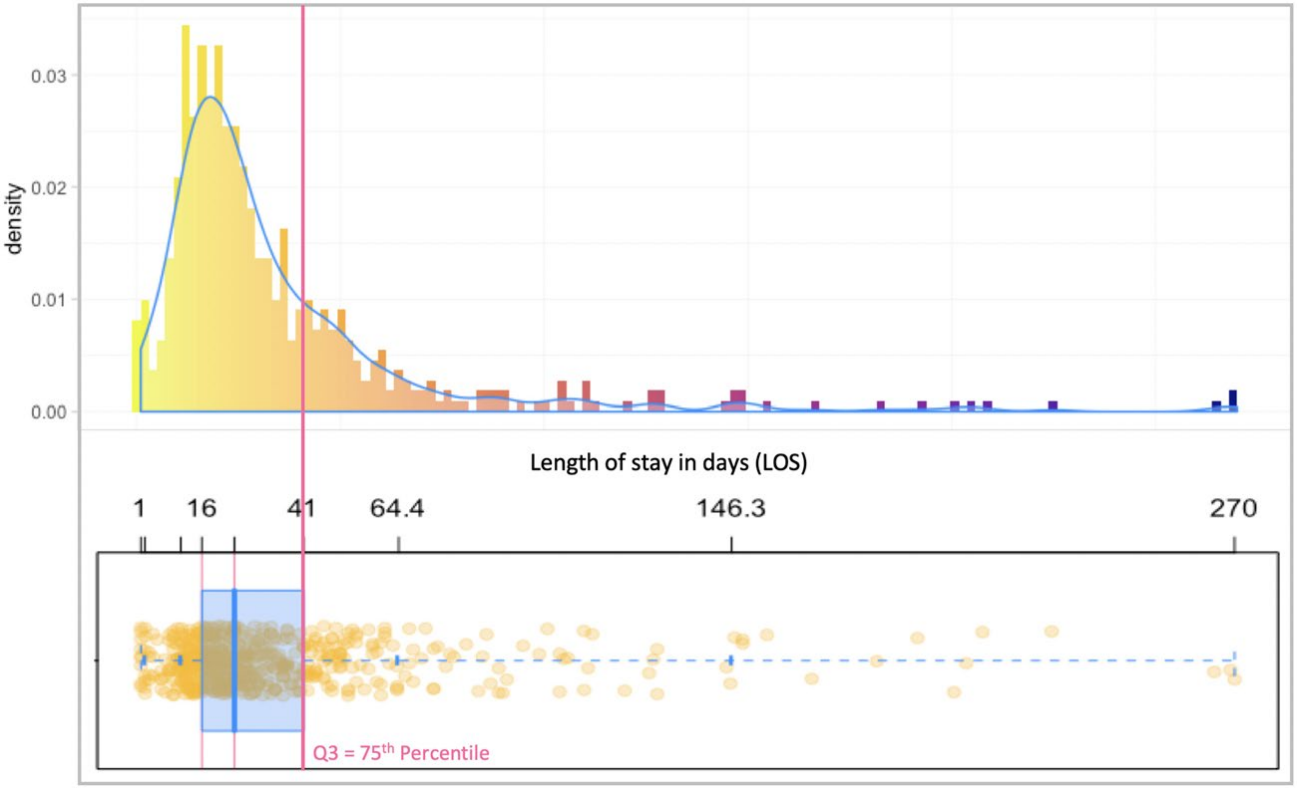
Histogram distribution and box plot of of the LOS data for patients who survived the Norwood procedure.

In the second part of the study we predicted the risk of prolonged LOS for patients after the first stage of the Norwood procedure. The patient’s condition during surgery has clinical relevance in making predictions about LOS post-surgery, so we used intraoperative data, such as bypass and cross-clamp times, in addition to the preoperative data used for mortality prediction. We added all operative variables from the PHN SVR dataset, making a total of 49 variables. Table 1 shows the 24 additional operative variables. To determine the threshold for prolonged LOS, we used the 75th percentile value proposed by previous PHN SVR studies^20,21^. As shown in Fig. 2, the 75th percentile for LOS for this dataset is 41 days.

The operative dataset was complete with no missing values and therefore, unlike the incomplete preoperative dataset, did not require imputation. We combined the intraoperative data with each of the 50 preoperative datasets created during the previous MCMC imputation process and ran the machine learning models on each of these aggregate datasets.

#### Model selection and training

We trained five machine learning models to predict risk of mortality or cardiac transplantation and prolonged length of stay: Ridge logistic regression, decision tree, random forest, gradient boosting, and deep neural network. We used Python scikit-learn^35^ and Keras^36^, which contain implementations of all models used in this study, to execute all aspects of model training and model evaluation. Sequential function was used to create the neural network model. RandomForestClassifier and GradientBoostingClassifier functions were used to create the random forest and gradient boosting classifiers respectively. Finally, LogisticRegression and DecisionTreeClassifier were used to create the logistic regression and decision tree classifiers.

Logistic regression has been extensively used in the medical field for the purpose of classification and regression. In logistic regression we use logit or sigmoid function to map the inputs to the output. Ridge logistic regression (LR) uses the square root of coefficient weights to prevent these coefficients from becoming very large during model training hence prevent overfitting of the model to the training data.

The decision tree (DT) model applies sequential partitioning technique to build a tree-based structure for generating a set of “if-then-else” rules in order to predict the outcomes^37,38^. The decision tree induction consists of two phases: construction and pruning. In the construction phase we progressively find a pair of an input and a threshold that splits the data to have maximum separation between the classes. In the pruning phase, we remove branches from the tree based on their lack of statistical significance. DT methodology is easily explainable since it provides a simple set of if-then-else rules that are actionable and hence widely understood and accepted in clinical practice. With complex datasets however, DT usually performs poorly when compared with other more sophisicated machine learning methods^38^.

Random forest (RF), is an ensemble learning method, uses the concept of bootstrap aggregating (bagging), averaging the predictions over bootstrap samples which are random samples of the dataset with replacement of used samples. This average is simply the majority vote of the classifiers for each sample. The observations in bootstrap samples are used for building a set of trees and all the observations that are not in these subsets are used for evaluating model performance^39^. Ensemble learning methods combine several machine learning classifiers, which are called base learners or weak learners, to achieve a better accuracy. The base learner for RF method is DT. DT algorithms use information gain to split a variable.

Gradient boosting (GB) is another ensemble learning method. At each step, a new weak or base-learner model is trained with respect to the error of the whole ensemble learned so far. In GB, the learning procedure consecutively fits new models to provide a more accurate estimate of the response variable. The base learner is again DT as at each iteration the GB method tries to fit a DT on the residual error from the previous step. The principle idea behind this algorithm is to construct the new base-learners to be highly correlated with the negative gradient or differentiation of the loss function associated with the whole ensemble^40^. Loss function is a measure of the error in prediction of outcome which is used to train or update the parameters of the machine learning model.

Neural networks have been extensively used in medical diagnostics including to predict ICU mortality and hospital length of stay^41–48^. Deep learning neural networks or deep neural networks (DNN) are specific types of neural networks that have more hidden layers and more neurons in each layer than traditional neural networks. They are able to achieve accurate results with highly varied data due to their ability to incorporate many parameters, enabling modeling of highly complex nonlinear systems such as those found in medicine.

### Bayesian optimization for hyperparameter tuning

Machine learning algorithms are parameter-heavy, which requires careful tuning of learning parameters and model hyperparameters such as number of neurons and number of layers. Unfortunately, this tuning often requires expert experience, rules of thumb, and sometimes includes manual search. There is therefore great appeal for the automation of hyperparameter selection and the tuning process. This approach can optimize the performance of deep neural networks to the problem at hand^49^.

Bayesian optimization method seeks to find the optimum of function $f(\theta )$ with regard to .. in a bounded set Θ by constructing a probabilistic model of $f(\theta )$ and then exploiting this model to adjust $\theta$.

We defined ${\theta }_{i}$ as the ${i}_{t}h$ sample and $f({\theta }_{i})$ as the observation of the objective function at ${\theta }_{i}$. We then defined the set of the observations as $D=\{{\theta }_{i},f({\theta }_{i})\}$. The prior distribution was combined with the likelihood function $P(D|f)$. We assumed that the $f(\theta )$ is drawn from a Gaussian probability distribution $P(f)=N(\mu ,\nu )$. Using the Bayes rule we then obtained the posterior distribution which captures our updated beliefs about the unknown objective function $f(\theta )$ as $P(f|D)\propto P(D|f)P(f)$.

To choose the next point to query, we must define an acquisition function, which quantifies the accuracy of a candidate solution. In this paper we used expected improvement (EI) as an acquisition function. $EI=E[max(\gamma -f(\theta ),0)]$ where, $\gamma$ is the best value of function so far. We continued the search until the EI in any direction was zero. In this study we used Bayesian optimization with EI for model tuning and finding the best parameters of machine learning algorithms. The best set of hyper-parameters for each model was selected based on the average performance of that model over all of the training and validation folds.

#### Design parameters

For assessing risk of mortality and prolonged length of stay, we optimized the weight decay coefficient and type of the computational routine solver for the Ridge regression model. The optimal set of hyperparameters of the machine learning models based on Bayesian optimization method strategy is presented in Table 2. In random forest we optimized the number of trees, criterion for splitting the trees, maximum depth of each tree and maximum number of features required to build each tree. We optimized the same hyperparameters as for the DT model, with the exception of number of estimators.

**Table 2:** Optimal parameters of the developed models based on the Bayesian optimization technique.

Model	Parameter	Range	Mortality	LOS
DNN	First hidden layer size	[100–200]	120	110
	Second hidden layer size	[80–180]	100	100
	Third hidden layer size	[20–70]	30	40
	Dropout ratio	[0.2–0.6]	0.5,0.5,0.2	0.5,0.5,0.2
	$\alpha$	[0.1–0.4]	0.2	0.1
	${\beta }_{1}$	[0.5–0.8]	0.5	0.6
	${\beta }_{2}$	[0.85–0.95]	0.9	0.9
	${\lambda }_{1}$	[0.001–0.005]	0.001	0.001
	${\lambda }_{2}$	[0.001–0.005]	0.001	0.001
GB	No of trees	[100–200]	160	130
	Learning rate	[0.1–0.3]	0.09	0.15
	Maximum depth	[3–7]	5	4
	Stochastic?	[Yes/No]	Yes	Yes
RF	No of trees	[100–200]	120	150
	Criterion	[Gini/Entropy]	Gini	Entropy
	Maximum depth	[3–7]	6	6
	Maximum features	[10–22]	13	21
DT	Criterion	[Gini/Entropy]	Gini	Gini
	Maximum depth	[3–7]	7	5
	Maximum features	[10–22]	11	22
LR	${L}^{2}$ weight	0.17	0.19	
	Solver	[Stochastic Average Gradient (SAG)/Newton]	SAG	SAG

The network size is the number of neurons in each layer. Dropout technique only applies to the hidden layers.

We used all the features to build each tree but used Bayesian optimization to find the maximum depth for trees. We also used Bayesian optimization to find the best number of DT to use in the model. The GB method is prone to overfitting to the training dataset so we applied a weighting factor for the residual error corrections by new trees when added to the model. This weighting factor is called learning rate and we used Bayesian optimization to find the best learning rate.

Finally, we designed DNNs with 3 hidden layers. We used Bayesian optimization to find the best set of hyperparameters for the neural network including the number of neurons in the hidden layers and the dropout ratio for each layer. We applied rectified linear activation function (ReLU) on the hidden layers and softmax activation function on the output layer. We used elastic net approach for regularizing the neural network^50^. In this method we modify the loss function by applying a linear combination of ${L}^{1}$ and ${L}^{2}$ regularization of neural network weights to avoid over-fitting of the function approximation. We used a binary cross-entropy measure between true and predicted output as the loss function. We use Adam optimization, which is an extension to stochastic gradient descent for training of the neural network^51,52^. We used Bayesian optimization to find learning rate ($\alpha$), hyper-parameters that control the decay rate of first (${\beta }_{1}$)- and second-order (${\beta }_{2}$) moments. For neural network weight initialization, we used the Xavier initialization method^53,54^. To avoid saturating nonlinearities in training the neural network, we used batch normalization.

### Model evaluation and validation

In order to evaluate and compare model performances we plotted the receiver operating characteristic (ROC) curve and also calculated the AUROC, accuracy and F-score values for each model. The ROC curve plots the true positive rate (TPR), also known as sensitivity, versus the false positive rate (FPR), or 1-specificity, at various threshold selections. AUROC is equal to the probability that a classifier will rank a randomly chosen positive instance higher than a randomly chosen negative one. The F-score is the harmonic mean of precision (positive predictive value) and recall (sensitivity) and is considered the most important performance measure for a machine learning model as it incorporates a measure of precision and recall values.

Although the 549 patient records from the PHN SVR dataset are an acceptable volume of data to train unbiased machine learning models, it is still a limited number and hence the traditional technique of dividing the data into training and testing sets may not yield sufficiently accurate and unbiased results. We therefore used 5-fold stratified cross validation for training, testing and validation of the model. Cross-validation is a statistical resampling procedure that is useful for evaluating the performance of a model over a limited data sample size. In this technique, we first shuffled the dataset randomly and split it into $k$ subsets, also called folds, then held one subset back as a test group and trained the model with the remaining folds. We repeated this process with different folds held back until all folds have been used as the test group. The reported accuracy measure is the mean of the calculated accuracy measure for each fold. We stratified each fold to have a similar patient survival ratio to ensure that the classifier performs consistently across the entire data-set.

#### Clustering

We performed clustering analysis, grouping patients from the PHN SVR dataset based on common features contributing to their risk of mortality. We selected the unsupervised machine learning k-means algorithm. We explored solutions ranging from 2–8 clusters and examined their Pseudo-F statistic- this compares the within-cluster with the between-cluster sum-of-squares. We then used these clusters with the most accurate machine learning model and data visualization software (Tableau, Seattle, USA) to develop a precision medicine tool that can be used during real time preoperative consultations to calculate an individual patient s risk of one-year mortality or cardiac transplantation.

Clustering was used to help clinicians interpret the probability results from DNN in the form of a data visualization to identify the high-risk population for mortality or cardiac transplantation. For each record in the original dataset, a mortality probability risk was calculated. Clustering was applied to the full range of probabilities to discern cluster membership. Thus, when a prediction is created for a new patient using the DNN model, the obtained probability score can be easily interpreted using cluster membership. Since this is a one-dimensional (nonhierarchical) data set cluster analysis, the number of clusters had to be pre-defined (k = 3) and was chosen based on practical constrains defined by the subject matter experts (cardiac surgeons and cardiologists) as low, medium and high risk.

## Results

### Mortality or Cardiac Transplantation Prediction

The best results for the individual risk of mortality or cardiac transplantation calculation were produced by the DNN model, which demostrated $89 \% \pm 4 \%$ accuracy, F-score of $0.89\pm 0.03$, and AUROC $0.95\pm 0.02$. In comparison, the AUROC result reported by Gupta *et al*.^11^, who used Bayesian Lasso regression modelling and 10-fold cross-validation, was 0.77. Chowdhury *et al*.^10^ achieved an AUROC of 0.79 using traditional statistical methods. These results show a superior discriminative ability of our DNN model for predicting outcomes in the PHN SVR dataset.

**Figure 3:**
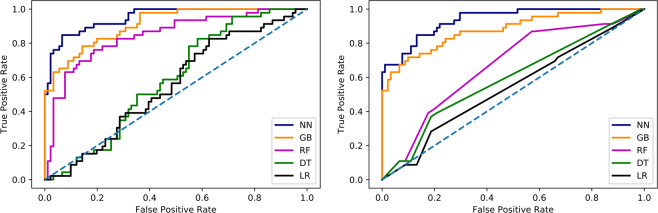
ROC curves afor each machine learning model after testing using all of the 50 MCMC MI datasets. (left) Risk of mortality prediction (right) Prolonged LOS prediction.

The detailed results of testing the machine learning models for prediction of mortality are presented in Table 3. Overall, for the mortality risk DNN classifier the accuracy of prediction is $89 \% \pm 4 \%$ and the AUROC is $0.95\pm 0.02$. The results for the gradient boosting model show $84 \% \pm 4 \%$ accuracy and AUROC of $0.90\pm 0.04$ which therefore also outperforms previously reported models. The random forest classifier has $75 \% \pm 5 \%$ accuracy and AUROC of $0.84\pm 0.03$. The F-score result of the random forest classifier is $0.43\pm 0.03$, showing reduced accuracy when compared with the DNN and GB models. This is mostly due to the low recall value of 0.27. The decision tree and Ridge regression models underperform in prediction of risk of mortality as they show AUROC of $0.58\pm 0.04$ and $0.55\pm 0.03$ respectively. Figure 3a shows the ROC curves associated with the lowest, median and highest AUROC for each machine learning model after training using all of the 50 datasets created during missing value MCMC MI procedure.

**Table 3:** Detailed results of machine learning classifiers for prediction of mortality and post-surgery prolonged LOS.

Model	Precision	Recall	F-Score	Accuracy	AUROC
**Mortality Prediction**					
Deep Neural Network	0.94 ± 0.03	0.86 ± 0.04	0.89 ± 0.03	0.89 ± 0.04	0.95 ± 0.02
Gradient Boosting	0.87 ± 0.03	0.78 ± 0.04	0.83 ± 0.04	0.84 ± 0.04	0.90 ± 0.04
Random Forest	0.71 ± 0.04	0.27 ± 0.03	0.43 ± 0.03	0.75 ± 0.05	0.84 ± 0.03
Decision Tree	0.43 ± 0.04	0.14 ± 0.05	0.29 ± 0.06	0.65 ± 0.04	0.58 ± 0.04
Ridge Regression	0.43 ± 0.04	0.10 ± 0.04	0.28 ± 0.03	0.61 ± 0.04	0.55 ± 0.03
**Prolonged LOS Prediction**					
Deep Neural Network	0.85 ± 0.04	0.91 ± 0.04	0.89 ± 0.04	0.85 ± 0.03	0.94 ± 0.04
Gradient Boosting	0.87 ± 0.04	0.82 ± 0.05	0.83 ± 0.03	0.82 ± 0.03	0.88 ± 0.03
Random Forest	0.62 ± 0.03	0.51 ± 0.05	0.55 ± 0.04	0.61 ± 0.03	0.67 ± 0.03
Decision Tree	0.56 ± 0.04	0.49 ± 0.04	0.52 ± 0.05	0.53 ± 0.04	0.59 ± 0.05
Ridge Regression	0.59 ± 0.05	0.32 ± 0.04	0.35 ± 0.04	0.63 ± 0.06	0.54 ± 0.07

We conducted the Friedman rank sum test^55^ to test the null hypothesis that all models have equal AUROC distributions over the cross-validation folds, which was rejected with ($p < 0.01$). We also used the student t-test to compare differences between model pairs. Our results show that DNN is statistically significantly better than GB ($p < 0.05$) and also all other models ($p < 0.01$). We also found that GB is statistically significantly better than all other models ($p < 0.01$).

### Prolonged length of stay prediction

The best results for individual risk of prolonged length of stay calculation was produced by the DNN model, demonstrating 85% accuracy, F-score of 0.89, and 0.94 AUROC. To the best of our knowledge, none of the previously published studies based on the PHN SVR dataset have attempted to predict length of stay.

The detailed results of testing the machine learning models for prediction of risk of prolonged LOS are presented in Table 3. Overall, the accuracy of the DNN prediction is $85 \% \pm 3 \%$ and the AUROC is $0.94\pm 0.04$. The GB model demonstrated $82 \% \pm 3 \%$ accuracy and AUROC of $0.88\pm 0.03$. The random forest, decision tree and Ridge regression models underperform in prediction of risk of prolonged LOS as they show AUROC of $0.67\pm 0.03$, $0.59\pm 0.05$ and $0.54\pm 0.07$ respectively. Figure 3b shows the ROC curves for each machine learning model after training using all of the 50 datasets created during missing value MCMC MI procedure.

We conducted the Friedman rank sum test^55^ to test the null hypothesis that all models have equal AUROC distributions over the cross-validation folds, which was rejected with ($p < 0.01$). We also used the student t-test to compare differences between model pairs. Our results show that DNN is statistically significantly better than GB ($p < 0.05$) and also all other models ($p < 0.01$). We also found that GB is statistically significantly better than all other models ($p < 0.01$).

### Clustering

Since this is a one-dimensional (nonhierarchical) data set cluster analysis, the number of clusters can either be driven by external considerations i.e. previous knowledge or known practical constrains or by different values for k. In our case, the k value was defined as 3 by the subject matter experts (cardiac surgeons, cardiologists) as “low risk”, “medium risk”, and “high risk” based on clinical factors such as observed vs. expected mortality cases.

Additionally, as the practice of clustering is known to be exploratory in nature, the authors wanted to substantiate that the intuition for the predefined value of k was suitable with the established methods to measure the correct segmentation using the Pseudo-F statistic values. The authors found that the F-score was suitable and within the range limits for cluster segmentation validity. Please refer to Table 4 for Pseudo-F score values.

**Table 4:** Detailed results clustering F-statistics.

Number of Clusters	F-Statistics
2	369.5
3	228.1
4	163.7
5	127.3
6	102.6
7	85.5
8	74.77

## Discussion

We investigated the use of machine learning techniques in the risk prediction for one-year mortality or cardiac transplantation in neonates for whom Norwood procedure was performed and risk of prolonged hospital LOS post-surgery. In this analysis, we used data provided by the PHN SVR trial to develop computational risk factor modeling and to produce an individualized risk calculator for clinical use.

We can predict, using a deep neural network model, patient-specific risk of one-year mortality or cardiac transplantation with $89 \% \pm 4 \%$ accuracy, F-score of $89 \% \pm 3 \%$ and an AUROC of $0.95\pm 0.02$. This outperformed the next best, gradient boosting, model by 0.05 in terms of AUROC and 6% in terms of f-score (..-value < 0.05). Compared to the most recently published study^11^, the results from our DNN model show a notable improvement of 0.22 in AUROC.

The results from prediction of prolonged hospital LOS using our DNN classifier show an accuracy of $85 \% \pm 3 \%$, AUROC of $0.94\pm 0.04$ and F-score of $89 \% \pm 4 \%$, indicating a highly robust model. Gradient boosting model also performed well in prediction by having an accuracy of $82 \% \pm 3 \%$, AUROC of $0.88\pm 0.03$ and F-score of $0.83\pm 0.03$. Clinicians and families will benefit from an accurate prediction by being able to plan the best care pathway during hospitalization and to prepare for the appropriate length of stay in the hospital. Nursing and administrative teams will also be able to better plan staffing and resources.

The random forest, decision tree, and logistic regression models had notably poor performance in comparison with DNN and gradient boosting methods, especially in the prediction of prolonged LOS. The poor performance of the decision tree and logistic regression models could be attributed to the fact that these methods are not designed to model the very complex nonlinear relationships that exist in the data. The random forest is based on the voting scheme between base learners where the predicted class is the predicted class by the majority of the base learners. The voting based models are generally prone to the bias induced by class imbalance.

Once the child receives the Norwood procedure, they are committed to a route of treatment that will in time incorporate the Glenn procedure, unless they die before Glenn is carried out. Therefore we aimed to provide a risk prediction for one year all-cause mortality or cardiac transplantation that can be used to inform pre-Norwood decision making. We could produce a model to predict mortality prior to Glenn procedure, but given the inevitability of the Glenn procedure after Norwood and also that Glenn carries a much smaller risk of death (148 pre-Glenn deaths vs. 17 pre-Fontan deaths^18^), we do not think that the accuracy of our model will be greatly affected, nor that controlling for complications from Glenn will add clinical relevance, as it will not affect the model’s primary benefit which is to predict long term survival prior to Norwood procedure. We used transplant-free survival at one year, rather than survival to completion of Fontan procedure, because this was the primary outcome for the PHN SVR study.

**Figure 4:**
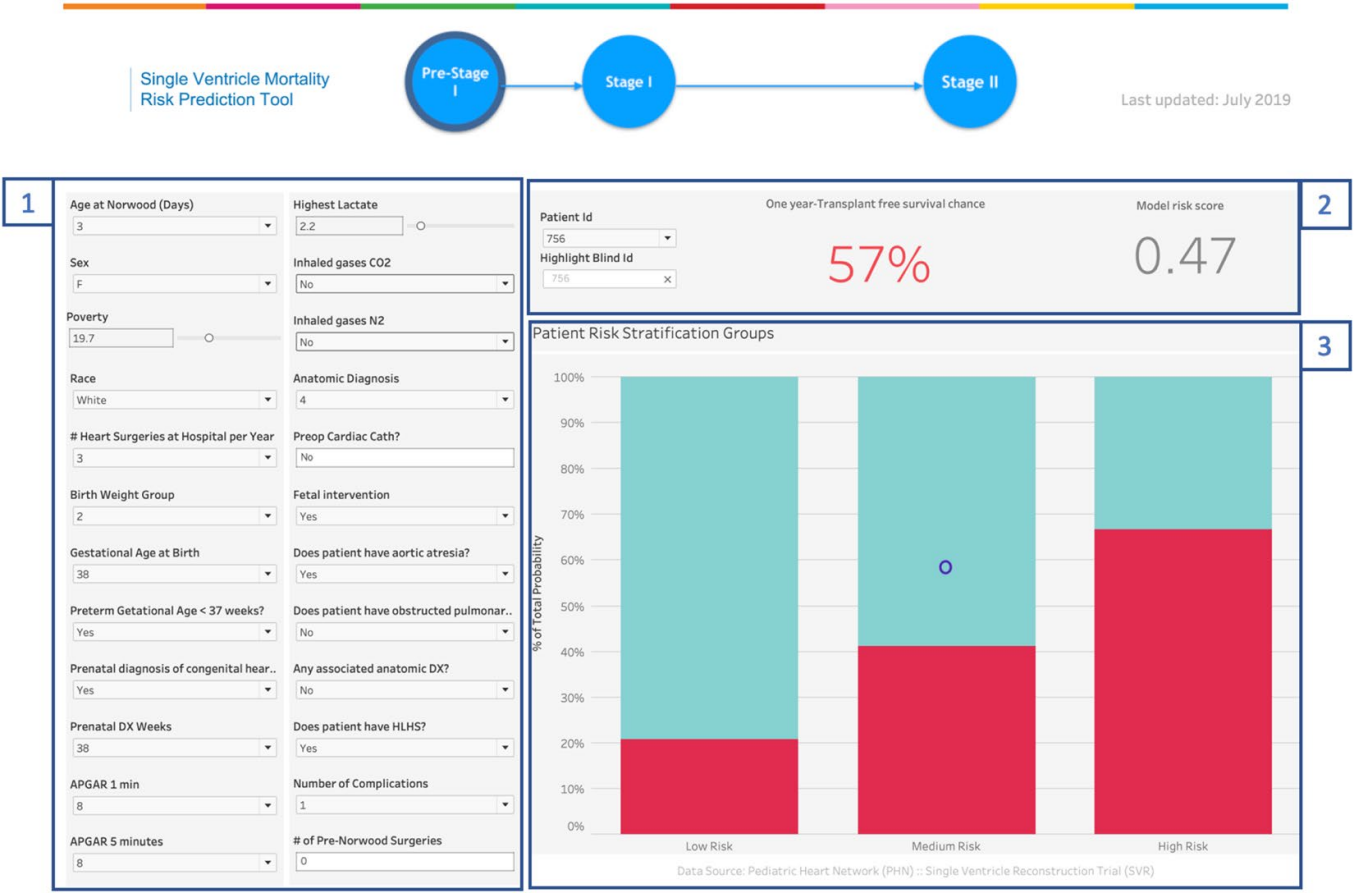
The calculator display of the mortality or cardiac transplantation risk and cluster for an example patient. Section labeled 1 contains two columns that allows the user to input a new patient’s data values such as age, sex, race, anatomic diagnosis etc. These values are used by the DNN model to calculate the patient specific risk score and provide a prediction for one-year transplant free survival (section 2). Section 3, a stacked bar graph, is used to depict cluster segments for the registry’s population risk scores and allow clinicians to evaluate a patient’s risk as low, medium or high.

As a future use for the developed model, we developed a precision medicine tool that can be used during real time preoperative consultations to calculate an individual patient s risk of one-year mortality or cardiac transplantation by using the DNN machine learning model with clustering analysis and data visualization software (Tableau, Seattle, USA). The end-user clinician enters routinely collected data for the 25 variables identified by our methodology from the PHN SVR dataset. Using these inputs and the DNN model, the calculator displays the current patient’s risk score. This output is then compared to a plot of pre-calculated PHN SVR patient risk scores to find the total number of patients with that same score who survived, and the number who did not. These two figures are then used to calculate and display the current patient’s probability of one year survival. To increase clinical utility and improve communication with families, our calculator also displays where the patient lies within the low, medium and high risk clusters, giving a visual representation of “patients like me”. Figure 4 shows the calculator display of the mortality or cardiac transplantation risk and cluster for an example patient from the PHN SVR dataset.

There are important limitations to our current models. Machine learning approaches and specifically deep learning methods generally require a large number of samples to properly train a model. Although the PHN SVR dataset is the largest publicly available dataset of its kind, and despite our use of k-fold technique, it’s relatively small size in machine learning terms could limit the performance of the models and introduce variance in model performance for use on new, prospective data. The PHN SVR trial only included patients that were considered fit enough for enrollment and excluded patients with complex anatomy or life-threatening comorbidities. This means that very sick patients with a consequent higher risk of mortality were excluded from the study, making the available dataset, and therefore any model, biased toward patients with higher survival rates. To maximize enrollment, the PHN SVR study was designed to have limited measurements in addition to simple, routinely collected information. As a result, more granular data such as parametric vital signs were not part of the required dataset. The variation in the machine learning models accuracy could be due to lack of more informative data. Another limitation is that the dataset is retrospective and hence we do not have access to truly unseen data to assess the performance of the machine learning models. Despite applying MCMC MI, one of the best methods to deal with missing values, the presence of missing values in the dataset may also lead to a biased model.

Statistical methods used to gain accurate insights into “big” datasets- those of large size and detailed granularity- have become more complex, requiring powerful computer algorithms and a knowledge of programming languages to fully understand and apply the techniques. This use of artificial intelligence may limit the ability to justify predictions in terms that clinicians understand and that they can trust. There is a need felt by clinicians to understand the relationships proposed by the model in order to target interventions, modify risk factors and facilitate shared decision-making with patients, families and the wider team. To satisfy this need, various recent attempts have been made to produce “explainable” machine learning models^56,57^.

A desire for clinical interpretability can come at the expense of model accuracy- our study demonstrates that the most accurate model is a deep neural network, a model that cannot be interrogated for individual contributing patient factors. Other models such as gradient boosters and random forest can be interrogated, for example to stratify risk factors; our study shows that, at least for this clinical question, these models are also less accurate. The individual patient parameters used in our study for prediction are mostly fixed and cannot be modified by clinical intervention at the point of preoperative decision-making. Therefore we felt it appropriate to use the most accurate model and to sacrifice an “explainable” element that would only be possible by using a different, less accurate model. This is a balance that physicians and data scientists will encounter more and more frequently as machine learning based decision support enters routine clinical practice.

## Conclusion

Our DNN model has improved accuracy over previously published attempts to predict risk of mortality or cardiac transplantation on an individual, rather than population level for neonates undergoing the Norwood surgical procedure. Accurate prediction of risk of mortality or cardiac transplantation and prolonged LOS based on preoperative data will facilitate a more informative discussion with families regarding the one year outcome after surgery for their child. Through the real-life application of this model and after further validation with prospective data, we hope to improve clinical decision making and give each family the best predictive information possible.

## Data Availability

The datasets analyzed during the current study are available in the Pediatric Heart Network repository, http://www.pediatricheartnetwork.org/ForResearchers/PHNPublicUseDatasets/SingleVentricleReconstruction-SVR.aspx.

## References

[1] Danford DA, Cronican P (1992). Hypoplastic left heart syndrome: progression of left ventricular dilation and dysfunction to left ventricular hypoplasia in utero. Am. heart J..

[2] Sano S (2003). Right ventricle–pulmonary artery shunt in first-stage palliation of hypoplastic left heart syndrome. J. Thorac. cardiovascular Surg..

[3] Takeuchi M (2003). Right ventricle of patients undergoing congenital cardiac surgery differentially expresses haem oxygenase-1 and heat shock protein 70 genes. J. Int. Med. Res..

[4] Dean PN, Hillman DG, McHugh KE, Gutgesell HP (2011). Inpatient costs and charges for surgical treatment of hypoplastic left heart syndrome. Pediatrics.

[5] Martin, J. A., Hamilton, B. E. & Osterman, M. J. Births in the united states, 2018. *NCHS Data Brief* (2019). 31442195

[6] Rychik J (2010). Perinatal and early surgical outcome for the fetus with hypoplastic left heart syndrome: a 5-year single institutional experience. Ultrasound Obstet. Gynecol..

[7] Ohye RG (2010). Comparison of shunt types in the norwood procedure for single-ventricle lesions. N. Engl. J. Med..

[8] Pasquali SK (2012). Variation in perioperative care across centers for infants undergoing the norwood procedure. J. Thorac. cardiovascular Surg..

[9] Tabbutt S (2012). Risk factors for hospital morbidity and mortality after the norwood procedure: a report from the pediatric heart network single ventricle reconstruction trial. J. Thorac. cardiovascular Surg..

[10] Chowdhury, S. M. *et al*. Validation of a simple score to determine risk of hospital mortality after the norwood procedure. In *Seminars in thoracic and cardiovascular surgery*, vol. 28, 425–433 (Elsevier, 2016). 10.1053/j.semtcvs.2016.04.004 PMC5214604 28043455

[11] Gupta P, Chakraborty A, Gossett JM, Rettiganti M (2017). A prognostic tool to predict outcomes in children undergoing the norwood operation. J. Thorac. cardiovascular Surg..

[12] Tu, J. V. & Guerriere, M. R. Use of a neural network as a predictive instrument for length of stay in the intensive care unit following cardiac surgery. *Proceedings. Symposium on Computer Applications in Medical Care* 666–672 (1992). PMC2248140 1482955

[13] Tu JV, Guerriere MR (1993). Use of a neural network as a predictive instrument for length of stay in the intensive care unit following cardiac surgery. Computers Biomed. research, an. Int. J..

[14] Widyastuti Y, Stenseth R, Wahba A, Pleym H, Videm V (2012). Length of intensive care unit stay following cardiac surgery: is it impossible to find a universal prediction model?. Interact. cardiovascular Thorac. Surg..

[15] Almashrafi A, Elmontsri M, Aylin P (2016). Systematic review of factors influencing length of stay in icu after adult cardiac surgery. BMC health Serv. Res..

[16] Ettema, R. G. A. *et al*. Prediction models for prolonged intensive care unit stay after cardiac surgery: systematic review and validation study. *Circulation* **122**, 682–9, 7 p following p 689 (2010). 10.1161/CIRCULATIONAHA.109.926808 20679549

[17] Messaoudi N, De Cocker J, Stockman B, Bossaert LL, Rodrigus IER (2009). Prediction of prolonged length of stay in the intensive care unit after cardiac surgery: the need for a multi-institutional risk scoring system. J. Card. Surg..

[18] Schwartz SM (2014). Risk factors for prolonged length of stay after the stage 2 procedure in the single-ventricle reconstruction trial. J. Thorac. cardiovascular Surg..

[19] Ravishankar C (2016). Factors affecting fontan length of stay: results from the single ventricle reconstruction trial. J. Thorac. cardiovascular Surg..

[20] Baker-Smith CM (2014). Predictors of prolonged length of intensive care unit stay after stage i palliation: a report from the national pediatric cardiology quality improvement collaborative. Pediatric cardiology.

[21] Baker-Smith CM, Goldberg SW, Rosenthal GL (2015). Predictors of prolonged hospital length of stay following stage ii palliation of hypoplastic left heart syndrome (and variants): Analysis of the national pediatric cardiology quality improvement collaborative (npc-qic) database. Pediatric cardiology.

[22] Bacha E, del Nido P (2012). Introduction to the single ventricle reconstruction trial. J. Thorac. cardiovascular Surg..

[23] Ghanayem NS (2012). Interstage mortality after the norwood procedure: results of the multicenter single ventricle reconstruction trial. J. Thorac. cardiovascular Surg..

[24] Liu Y, De A (2015). Multiple imputation by fully conditional specification for dealing with missing data in a large epidemiologic study. Int. J. Stat. Med. Res..

[25] Ni, D. & Leonard II, J. Markov chain monte carlo multiple imputation using bayesian networks for incomplete intelligent transportation systems data. *Transportation Research Record: Journal of the Transportation Research Board* 57–67 (2005).

[26] Brooks, S., Gelman, A., Jones, G. & Meng, X.-L. *Handbook of markov chain monte carlo* (CRC press, 2011).

[27] Oster ME, Kelleman M, McCracken C, Ohye RG, Mahle WT (2016). Association of digoxin with interstage mortality: results from the pediatric heart network single ventricle reconstruction trial public use dataset. J. Am. Heart Assoc..

[28] Sun, W. Application of markov chain monte-carlo multiple imputation method to deal with missing data from the mechanism of mnar in sensitivity analysis for a longitudinal clinical trial. In *Monte-Carlo Simulation-Based Statistical Modeling*, 233–252 (Springer, 2017).

[29] Yuan YC (2010). Multiple imputation for missing data: Concepts and new development (version 9.0). SAS Inst. Inc, Rockville, MD..

[30] Musoro JZ, Zwinderman AH, Puhan MA, ter Riet G, Geskus RB (2014). Validation of prediction models based on lasso regression with multiply imputed data. BMC Med. Res. Methodol..

[31] Wahl S, Boulesteix A-L, Zierer A, Thorand B, van de Wiel MA (2016). Assessment of predictive performance in incomplete data by combining internal validation and multiple imputation. BMC Med. Res. Methodol..

[32] Hughes R, Sterne J, Tilling K (2016). Comparison of imputation variance estimators. Stat. methods Med. Res..

[33] Buuren, S. V. & Groothuis-Oudshoorn, K. mice: Multivariate imputation by chained equations in r. *Journal of statistical software* 1–68 (2010).

[34] Chowdhury SM (2015). Validation of a simple score to determine risk of hospital mortality after the norwood procedure. Circulation.

[35] Pedregosa F (2011). Scikit-learn: Machine learning in python. J. Mach. Learn. Res..

[36] Chollet, F. Keras, https://keras.io (2015).

[37] Jalali, A., Licht, D. J. & Nataraj, C. Application of decision tree in the prediction of periventricular leukomalacia (PVL) occurrence in neonates after heart surgery. In *2012 Annual International Conference of the IEEE Engineering in Medicine and Biology Society*, 5931–5934 (IEEE, 2012). 10.1109/EMBC.2012.6347344 PMC3710705 23367279

[38] Jalali A (2013). Prediction of periventricular leukomalacia occurrence in neonates after heart surgery. IEEE J. Biomed. health Inform..

[39] Breiman L (2001). Random forests. Mach. Learn..

[40] Schapire, R. E. The boosting approach to machine learning: An overview. In *Nonlinear estimation and classification*, 149–171 (Springer, 2003).

[41] Haykin SS, Haykin SS, Haykin SS, Elektroingenieur K, Haykin SS (2009). Neural networks and learning machines.

[42] Amato F (2013). Artificial neural networks in medical diagnosis. J. Appl. Biomedicine.

[43] Hachesu PR, Ahmadi M, Alizadeh S, Sadoughi F (2013). Use of data mining techniques to determine and predict length of stay of cardiac patients. Healthc. Inform. Res..

[44] Cheng C-W, Chanani N, Venugopalan J, Maher K, Wang M (2013). D. icuarm-an icu clinical decision support system using association rule mining. IEEE J. Transl. Eng. health Med..

[45] Dabek, F. & Caban, J. J. A neural network based model for predicting psychological conditions. In *International Conference on Brain Informatics and Health*, 252–261 (Springer, 2015).

[46] Pirracchio, R. Mortality prediction in the icu based on mimic-ii results from the super icu learner algorithm (sicula) project. In *Secondary Analysis of Electronic Health Records*, 295–313 (Springer, 2016). 31314257

[47] Saha B, Gupta S, Phung D, Venkatesh S (2017). A framework for mixed-type multioutcome prediction with applications in healthcare. IEEE J. Biomed. Health Inform..

[48] Gálvez JA, Jalali A, Ahumada L, Simpao AF, Rehman MA (2017). Neural network classifier for automatic detection of invasive versus noninvasive airway management technique based on respiratory monitoring parameters in a pediatric anesthesia. J. Med. Syst..

[49] Jones DR (2001). A taxonomy of global optimization methods based on response surfaces. J. Glob. Optim..

[50] Zou H, Hastie T (2005). Regularization and variable selection via the elastic net. J. R. Stat. Society: Ser. B.

[51] Le, Q. V. *et al*. On optimization methods for deep learning. In *Proceedings of the 28th International Conference on International Conference on Machine Learning*, 265–272 (Omnipress, 2011).

[52] Kingma, D. P. & Ba, J. Adam: A method for stochastic optimization. *arXiv preprint arXiv:1412.6980* (2014).

[53] Glorot, X. & Bengio, Y. Understanding the difficulty of training deep feedforward neural networks. In *Proceedings of the thirteenth international conference on artificial intelligence and statistics*, 249–256 (2010).

[54] He, K., Zhang, X., Ren, S. & Sun, J. Delving deep into rectifiers: Surpassing human-level performance on imagenet classification. In *Proceedings of the IEEE international conference on computer vision*, 1026–1034 (2015).

[55] Masino, A. J. *et al*. Machine learning models for early sepsis recognition in the neonatal intensive care unit using readily available electronic health record data. *PloS one* **14** (2019). 10.1371/journal.pone.0212665 PMC6386402 30794638

[56] Lundberg SM (2018). Explainable machine-learning predictions for the prevention of hypoxaemia during surgery. Nat. Biomed. Eng..

[57] Lee H (2019). An explainable deep-learning algorithm for the detection of acute intracranial haemorrhage from small datasets. Nat. Biomed. Eng..

